# Reliability of the 30 s Chair Stand Test in Women with Fibromyalgia

**DOI:** 10.3390/ijerph16132344

**Published:** 2019-07-02

**Authors:** Juan Pedro Martín-Martínez, Daniel Collado-Mateo, Francisco Javier Domínguez-Muñoz, Santos Villafaina, Narcís Gusi, Jorge Pérez-Gómez

**Affiliations:** 1 Faculty of Sport Science, University of Extremadura, Avda: Universidad S/N, 10003 Cáceres, Spain; 2 Facultad de Educación, Universidad Autónoma de Chile, Talca 1670, Chile

**Keywords:** intraclass correlation coefficient, standard error of measurement, chronic pain, reliability analysis, physical fitness tests

## Abstract

*Background:* The 30 s chair stand test is often used to evaluate physical fitness in chronic pain populations. In patients with fibromyalgia, physical fitness is closely related to pain, quality of life, and fear of falling. However, the reliability of this test has only been evaluated concerning the number of repetitions. *Objective:* To evaluate the test–retest reliability of the 30 s chair stand test in women with fibromyalgia (*n* = 30), using data from the contact and non-contact time registered with an automatic chronometer (chronojump). *Methods:* Participants carried out the 30 s chair stand test twice with five minutes as a rest period, while an automatic chronometer recorded the time elapsed in contact with the chair (impulse phase) and not in contact (non-contact phase). Number and fear of falls in the last year and in the last six weeks were also recorded. *Results*: The reliability of duration of both phases was good. A relationship between these results and the number and fear of falling was also found. *Conclusion*: The analysis of movement phases in the 30 s chair stand test showed a good reliability in females with fibromyalgia, providing further useful information about the onset of muscle fatigue during the test.

## 1. Introduction

Fibromyalgia (FM) is defined as a chronic disease characterized by widespread persistent pain and associated with other symptoms, such as non-restorative sleep, anxiety, depression, stiffness, fatigue, balance, and mobility problems [1,2]. Therefore, people affected by FM often have a reduced health-related quality of life [3], and experience difficulties in performing their daily activities [4].

The prevalence of FM depends on the country and the criteria. The modification of the American College of Rheumatology Preliminary Diagnostic Criteria for FM in 2010 has allowed researchers to determinate whether a patient is suffering from FM or not, outside of the clinical setting [5]. Using this criteria, the prevalence of FM is 1.8% in the United States [6]. In Europe, the prevalence ranges from 2.9 to 4% of the general population [7]. FM is 20 times more frequent among women than men, according to Mas et al. [8], who stated that FM affects more than 4% of Spanish women, while the prevalence among men is only 0.2%.

Physical fitness is an important measurement in patients with FM, since it is closely related to pain [9], quality of life [10], fear of falling [2], and psychological disorders [11]. Furthermore, the ability to perform daily activities is conditioned by physical fitness [12] and fear of falling [2]. In this regard, poor physical fitness, recent falls, or fear of falling, may cause avoidance of motor activities of daily living [13].

One of the most widely used test is the 30 s chair stand test [14], which involves the repetition of the sit-to-stand-to-sit pattern. The evaluation of the sit-to-stand-to-sit task provides relevant information in chronic pain populations [15]. In women with FM, the 30 s chair stand test has been used to discriminate the presence/absence of FM and the severity of the associated symptoms [16]. 

Although the number of repetitions in the 30 s chair stand test are closely related to the physical impact of FM and pain levels [17], previous research has suggested that the kinematics involved in performing this test may be even more relevant. In this regard, Collado-Mateo et al. [18] divided the task into two (sit-to-stand and stand-to-sit phases) and three phases (stand-up, sit down, and impulse phases) using the Functional Assessment of Biomechanics (FAB) device (Biosyn Systems, Inc.; Surrey, BC, Canada). They observed that the duration of phases was related to relevant FM-related variables, and suggested that division into either two or three phases provides useful information. However, the reliability of the duration of phases of the 30 s chair stand test in women with FM has never been reported. Given that the score achieved in a test is variable and may differ from one repetition to another performed in the same condition, this reliability information is necessary for clinicians and researchers in order to interpret changes in variables after a specific intervention.

A previous study [19] has evaluated the reliability of the 30 s chair stand test in women with FM. However, it was focused only on the number of repetitions, and, to our knowledge, no study has evaluated the reliability of the test taking into account the duration of the movement phases in each repetition. Furthermore, the FAB device is expensive, so we propose this analysis, focusing on the impulse phase (IP) and non-contact phase (NCP), using a cheaper instrument: the Chronopic (Chronojump).

Therefore, the main objective of this study was to evaluate the reliability of two phases of the 30 s chair stand test in women with FM, using data from the contact time registered with the Chronojump device.

## 2. Materials and Methods

### 2.1. Participants

A total of 30 women from a local association of FM took part in this study. Their main characteristics are shown in Table 1. A total 73.33% of the participants were overweight or obese. Furthermore, 40% and 33% of the participants were under antidepressant and analgesic treatments respectively, and physiotherapy was the main complementary therapy (26.7% of the participants). They were required to fulfil the following inclusion criteria: (a) Have been diagnosed with FM by a rheumatologist according to the 2010 criteria established by the American College of Rheumatology [20]; (b) Be able to communicate effectively with the study staff; (c) Be older than 18 years old; and (d) Have understood and signed the informed consent in accordance with the updated Declaration of Helsinki. On the other hand, participants were excluded if they were not able to stand and sit on the chair once without help. This study obtained the agreement of the Biomedical Ethics Committee of the University of Extremadura (Spain) (62/2017).

### 2.2. Procedure

First, participants answered some socio-demographic questions, a question about the number of falls in the last year, as well as a visual analogical scale (VAS) about fear of falling ranging from 0 to 100, where 0 meant “no fear” and 100 meant “extreme fear,” which was validated by Scheffer et al. [21]. This VAS has been previously used in women with fibromyalgia [2,22]. They also completed the Fibromyalgia Impact Questionnaire [23,24], which evaluates the impact of the symptoms on fibromyalgia from 0 (minimum impact) to 100 (maximum impact). In Spain, this questionnaire was developed by Esteve-Vives et al. [25]. Then, participants were measured and weighed using the Tanita Body Composition Analyzer BC-418 MA.

After that, all the participants were informed of the essential instructions and considerations on the development of the 30 s chair stand test and performed one repetition in order to get used to it. Then, they completed the 30 s chair stand test twice with a rest period of five minutes between both attempts.

### 2.3. The 30 s Chair Stand Test

To perform the 30 s chair stand test, the participants began seated on a chair with their arms crossed at the chest level and the hands over their shoulders. They had to stand up from this seated position until they reached a complete knee extension, and then sit down again until the back touched the backrest of the chair. This cycle was repeated as many times as they could in 30 seconds. Before starting the 30 s chair stand test, a member of the study staff explained the protocol to the participants, encouraging them to maintain the arms crossed position throughout the test, as well as reaching a complete knee extension and touching the backrest of the chair when sitting down. Moreover, as a security measure, a member of the study staff was always holding the back of the chair in order to restrict its movement, and he was in charge of monitoring the time of the test.

### 2.4. Outcomes

Durations of IP and NCP were calculated by using the free software chrono-jump (Chronojump-BoscoSystem, Barcelona, Spain) with the open hardware Chronopic [26]. In our case, the Chronopic was placed on the chair seat. This automatic chronometer allows recording of the time elapsed when an electric circuit is opened (i.e., the participant was not touching the device, NCP), and when it is closed (i.e., the participant was in contact with the device, IP). The IP takes place when subjects are seated on the chair, and they began to stand in order to adopt a standing position, whereas the NCP starts at the moment when they leave the surface of the chair, and continues until the instant when participants touch the tape of Chronopic. This method is a valid way to record the time elapsed, since the circuit is opened until it is closed again [27].

### 2.5. Statistical Analysis

#### 2.5.1. Normality Analysis and Differences between Test and Retest

For the statistical analysis, Kolmogorov–Smirnov and Shapiro–Wilk tests were conducted. Based on the results from those tests, non-parametric statistical analysis was chosen. The Wilcoxon rank test was conducted to evaluate differences between test and retest in both the number of repetitions and the kinematic variables of the 30 s chair stand test, including the mean duration of IP and NCP during the 30 s, and also the mean duration of the IP in the first and last repetitions. This statistical analysis was also conducted to detect differences between the initial and the last repetition of the 30 s chair stand test both in the test and the retest.

#### 2.5.2. Reliability Analysis

Reliability was estimated using recommendations by Weir [28] concerning the intraclass correlation coefficient (ICC) random effects model analysis of variance. Furthermore, absolute reliability was established by calculating the standard error of measurement (SEM) following this formula:

(1) $$\mathrm{SEM}\;=\;\mathrm{SD}\;\sqrt{1-\mathrm{ICC}}$$

where SD is the mean standard deviation of the two repetitions. The smallest real difference (SRD) was calculated according to the formula:

(2) $$1.96\;\times \;\mathrm{SEM}\;\times \;\sqrt{2}$$

Both SEM and SRD are expressed in percentages in order to facilitate the comparability with previous studies.

#### 2.5.3. Correlation Analyses

Finally, Spearman’s rho correlation analyses were used to evaluate the relationship between fall-related variables (number of falls and fear of falling) and the mean duration and the duration of the initial and final phases in both IP and NCP.

## 3. Results

The main characteristics of the participants, including age, anthropometric measures, the impact of FM assessed using the Fibromyalgia Impact Questionnaire (FIQ), and years since FM was diagnosed are shown in Table 1.

Table 2 shows the means and SD of the total number of repetitions of test and retest performed by subjects, as well as the mean duration of IP and NCP during test and retest, and also the mean duration of IP and NCP at the first and last repetition in test and retest. Significant differences were found between test and retest in the total number of repetitions, the mean NCP duration, and the mean duration of IP and NCP in the last repetition comparing test and retest.

In order to explore the influence of the fatigue within the 30 s chair stand test, Table 3 included comparisons between the initial (the first repetition of the test) and the final repetition (the last repetition of the test) in both IP and NCP. There were no significant differences either in the test or in the retest assessment.

The reliability of the number of repetitions in the 30 s chair stand test was 0.876, which is good (>0.70 and <0.90) according to the classification by Munro et al. [29]. Regarding phase durations, we obtained an ICC of 0.866 (0.178–0.936) in the mean IP duration, and 0.929 (0.850–0.966) in the mean NCP duration. This last score represents an excellent (>0.90) reliability [29]. The %SEM was 6.28% for IP and 6.56% for NCP, which was slightly lower than the observed for the number of repetitions in the test. Finally, the SRD and %SRD are also reflected in Table 4 for both phase durations. Thus, we found a %SRD of 17.41% for IP, and 18.18% for NCP duration (see Table 4).

Finally, Table 5 shows the correlations between kinematic variables extracted from the 30 s chair stand test and fall-related variables. As can be observed, both the number of repetitions and kinematic variables were significantly related to fear of falling assessed using VAS, whereas no correlation with the number of falls was detected.

## 4. Discussion

The results of the study show that the reliability of the number of repetitions and the mean duration of the two phases of 30 s chair stand test was good or excellent in women with fibromyalgia. Interestingly, kinematic variables and the number of repetitions in the 30 s chair stand test were significantly related to fear of falling, but not to the number of falls. The current study is the first to evaluate the reliability of this test using a simple instrument that allows collection of data to determine the movement phases of the sit-to-stand-to sit task in women with fibromyalgia. Reliability analysis showed that duration of phases extracted using the chronopic device is reliable enough to warrant an appropriate assessment. However, in the case of the initial repetition, the low ICC value and the high %SEM and %SRD imply that the use and interpretation of these variables must be done with caution.

The reliability and feasibility of the 30 s chair stand test has already been evaluated in different populations, such as FM females [19], patients with stroke [30], or older adults with type 2 diabetes [31]. However, these authors focused on the reliability of the number of repetitions and, to our knowledge, no studies have investigated the different phases of movement in this test.

The ICC values for stand repetitions, the IP mean, and NCP mean were slightly lower than previous studies [19,30,31]. In this regard, we obtained 0.87, 0.86, and 0.92 as ICC, respectively. On the other hand, Carbonell-Baeza et al. [19] showed an ICC value of 0.91 in a similar study with FM female patients. One possible reason for this slight difference might be the participants’ age, since the subjects in our study were four years older. Other factors like the physical fitness of the women and/or their FM severity could also justify the differences. 

Concerning SEM, the results of this study are similar to those of previous studies [19,30,31], even lower, probably due to the precision of Chronopic with regard to the assessment of IP and NCP time. Apart from the SEM, the current study also reports the SRD, which states the minimal change that should be achieved to consider it as real. The %SRD were around 18% in the number of repetitions and in the mean duration of IP and NCP. However, the %SRD was higher than 20% when the variables were analyzed at the end of the task, and close to 40% when they were analyzed at the beginning of the task. Reliability analyses revealed that phase durations in the initial repetition was not reliable. Therefore, the mean duration of phases or the evaluation of durations at the end of the task are recommended.

Similar to the findings of Carbonell-Baeza et al. [19], familiarization or learning effects were observed in retest measures regarding test results (Table 2). In this regard, there was a significant increase in the number of repetitions in the retest compared with the test results. In line with this increase, there was a significant reduction of phase durations at the end of the task in both phases. Regarding the mean duration of phases, there was a reduction in both the IP and NCP, but it only reached significance in the NCP, while the p-value for the reduction of IP was 0.08. Thus, it could be possible that the motor pattern was different in the retest as a consequence of familiarization. However, future studies using motion capture techniques should investigate how the motor pattern could change during the 30 s chair stand test, and also how familiarization with the task and learning might modify it.

A previous study, conducted in older adults with a similar protocol, evaluated the test–retest reliability of the IP and NCP using the chronopic device [32]. They found that the number of repetitions and also the mean duration of IP and NCP phases were reliable (ICC higher than 0.80 and lower than 0.90), while the reliability of those variables when analyzing the beginning or the end of the 30 s chair stand test was not always good. Thus, taking into account both these results and those obtained in the present study, the evaluation of the duration of phases along the test must be used with caution. 

This study is also in line with the previous one using the chronopic device to extract phases from the sit-to-stand-to-sit cycle [32]. This instrument is a reliable low-cost alternative to obtain further information from a widely used test like the 30 s chair stand test. A previous study focused on sit-to-stand-to-sit evaluation in women with fibromyalgia used the Functional Assessment of Biomechanics (FAB) device, which is much more expensive [18]. The potential of the FAB device is higher, since it allows the extraction of more phases. In this regard, the mentioned previous study divided the NCP into two phases: stand-up and sit-down. Furthermore, they also divided the cycle into the sit-to-stand (beginning when the back is touching the backrest and ending when the standing position is reached) and the stand-to-sit (beginning in the stand position and ending when the back touched the backrest). However, given that, to our knowledge, no study has evaluated the test–retest reliability of the FAB device, reliability comparisons between the two instruments cannot be performed. Therefore, although the chronopic device is reliable for extracting phases, it must be noted that other devices may offer more alternatives to perform kinematic analyses.

Another interesting finding of the current study was that both the kinematic variables and the number of repetitions in the 30 s chair stand test were significantly related to fear of falling, but not to the number of falls. A previous study observed that perceived balance was related to fear of falling, while objective balance was associated with the number of falls [2]. However, in that article, the number of repetitions in the 30 s chair stand test was not significantly associated with either fear of falling (*p*-value = 0.08) or the number of falls (*p*-value = 0.93). The highest correlation coefficient was observed in the final NCP duration, which may reflect the onset of fatigue as a consequence of the execution of the task. Therefore, future studies may include this variable to evaluate kinematic performance in the 30 s chair stand test. Furthermore, that variable achieved excellent reliability (ICC > 0.90) in the current study with women suffering from fibromyalgia.

The current study has some limitations. First, the time of day in which the test was performed was not controlled, the sample size was relatively small, and some uncontrolled factors, including complementary therapies, muscle temperature, depression, or obesity, could affect the results. In this regard, 73.33% of the participants were overweight or obese, which is a usual profile among women with fibromyalgia [16]. Furthermore, data regarding the treatments that participants are receiving must be considered to appreciate the profile of the patients. Finally, although the analysis showed a good reliability, more studies are needed to check why there were significant differences between test and retest.

## 5. Conclusions

This is the first study that has evaluated the reliability of the duration of the movement phases in repetitions of the 30 s chair stand test with a low-cost instrument. The analysis of phase duration using the Chronopic instrument showed a good to excellent reliability in females with FM, except when the variables were analyzed at the beginning of the task. Thus, duration of phases extracted using the chronopic device is reliable enough to warrant an appropriate assessment. However, in the case of the initial repetition, the low ICC value and the high %SEM and %SRD imply that the use and interpretation of these variables must be taken with caution. Furthermore, the number of repetitions and durations of IP and NCP were significantly associated with fear of falling, but not with the number of falls.

## Figures and Tables

**Table 1:** Descriptive characteristics of the participants.

Participants	Mean ± SD
Sample size	30
Age (years)	54.8 ± 10.3
FIQ-80	43.3 ± 13.4
Year since initial diagnosis	11.0 ± 6.9
Height (cm)	160.1 ± 6.3
Weight (kg)	69.5 ± 10.9
BMI (kg/m^2^)	27.13 ± 4.15
BMI (kg/m^2^) classification (n of patients)	
18.5–24.9	8 (26.67%)
25.0–29.9	13 (43.33%)
30–34.9	7 (23.33%)
35–39.9	1 (3.33%)
Medication (n of patients)	
Antidepressants	12 (40%)
Analgesics/relaxants	10 (33%)
Complementary therapies (n of patients)	
Massages	5 (16.7%)
Physiotherapy	8 (26.7%)

FIQ: Fibromyalgia Impact Questionnaire; SD: Standard deviation.

**Table 2:** T-test between test and retest.

Measures	Test (Mean ± SD)	Retest (Mean ± SD)	*p*-Value	Cohen’s D
Total repetitions	11.37 ± 2.13	11.88 ± 2.25	0.016	0.275
Mean_IP	1.23 ± 0.22	1.18 ± 0.20	0.098	0.206
Mean_NCP	1.55 ± 0.37	1.47 ± 0.37	0.023	0.327
IP initial ^1^	1.20 ± 0.29	1.19 ± 0.25	0.713	0.113
IP final ^2^	1.23 ± 0.26	1.16 ± 0.18	0.041	0.469
NCP initial ^1^	1.53 ± 0.43	1.48 ± 0.31	0.262	0.144
NCP final ^2^	1.57 ± 0.42	1.49 ± 0.42	0.023	0.288

IP: impulse phase; NCP: non-contact phase; ^1^ IP and NCP time of the first repetition; ^2^ IP and NCP of the last repetition.

**Table 3:** Impulse phase and non-contact phase comparison between initial and final repetition in the test and retest.

Measures (s)	Initial (Mean ± SD)	Final (Mean ± SD)	*p*-Value	Cohen’s D
Test measures				
IP ^1^	1.20± 0.29	1.23 ± 0.26	0.688	0.141
NCP ^2^	1.53 ± 0.43	1.57 ± 0.42	0.711	0.09
Retest measures				
IP ^1^	1.19 ± 0.25	1.16 ± 0.18	0.544	0.108
NCP ^2^	1.48 ± 0.31	1.49 ± 0.42	0.537	0.033

IP: Impulse Phase; NCP: non-contact phase. ^1^ Differences between IP in first and last repetition; ^2^ Differences between NCP in first and last repetition.

**Table 4:** Reliability of 30 s chair stand test.

Variable	ICC (95% CI)	SEM	%SEM	SRD	%SRD
30 s chair Stand test	0.876 (0.755–0.939)	0.77	6.64	2.14	18.41
Mean_IP (s)	0.866 (0.718–0.936)	0.07	6.28	0.21	17.41
Mean_NCP (s)	0.929 (0.850–0.966)	0.09	6.56	0.27	18.18
IP_Initial (s)	0.596 (0.306–0.785)	0.17	14.34	0.47	39.77
NCP_Initial (s)	0.674 (0.419–0.830)	0.21	13.99	0.58	38.80
IP_Final (s)	0.726 (0.499–0.859)	0.11	9.81	0.32	27.20
NCP_Final (s)	0.902 (0.804–0.952)	0.13	8.54	0.36	23.68

ICC: intraclass correlation coefficient; SEM: standard error of measurement; SRD: smallest real difference. IP: impulse phase; NCP: non-contact phase.

**Table 5:** Relationship between fear of falling, number of falls, and other variables related to phases of movement in the 30 s chair stand test.

Variable		*N*	Number of Falls in the Last Year		Fear of Falling Last Year	
			Test	Retest	Test	Retest
Number of repetitions in the 30 s chair stand test	Correlation coefficient	30	−0.183	−0.006	−0.474 *	−0.375 *
	*p*-value		0.333	0.976	0.008	0.041
Initial impulse phase duration	Correlation coefficient	30	0.341	-0.031	0.306	0.458 *
	*p*-value		0.065	0.873	0.100	0.011
Mean impulse phase duration	Correlation coefficient	30	0.218	−0.011	0.422 *	0.366 *
	*p*-value		0.247	0.954	0.020	0.047
Final impulse phase duration	Correlation coefficient	30	0.179	0.129	0.387 *	0.430 *
	*p*-value		0.343	0.496	0.035	0.018
Initial non-contact phase duration	Correlation coefficient	30	0.172	0.018	0.380 *	0.306
	*p*-value		0.362	0.927	0.038	0.101
Mean non-contact phase duration	Correlation coefficient	30	0.137	−0.051	0.481 *	0.383 *
	*p*-value		0.470	0.789	0.007	0.037
Final non-contact phase duration	Correlation coefficient	30	0.112	−0.040	0.500 *	0.365 *
	*p*-value		0.555	0.835	0.005	0.048

* *p*-value lower than 0.05. For correlation analyses, Spearman’s rho was used.
